# Unsupervised learning to characterize patients with known coronary artery disease undergoing myocardial perfusion imaging

**DOI:** 10.1007/s00259-023-06218-z

**Published:** 2023-04-17

**Authors:** Michelle C. Williams, Bryan P. Bednarski, Konrad Pieszko, Robert J. H. Miller, Jacek Kwiecinski, Aakash Shanbhag, Joanna X. Liang, Cathleen Huang, Tali Sharir, Sharmila Dorbala, Marcelo F. Di Carli, Andrew J. Einstein, Albert J. Sinusas, Edward J. Miller, Timothy M. Bateman, Mathews B. Fish, Terrence D. Ruddy, Wanda Acampa, M. Timothy Hauser, Philipp A. Kaufmann, Damini Dey, Daniel S. Berman, Piotr J. Slomka

**Affiliations:** 1 Departments of Medicine (Division of Artificial Intelligence in Medicine), Biomedical Sciences, and Imaging, Cedars-Sinai Medical Center, 8700 Beverly Boulevard, Ste. Metro 203, Los Angeles, CA 90048 USA; 2 British Heart Foundation Centre for Cardiovascular Science, University of Edinburgh, Edinburgh, UK; 3 Department of Cardiac Sciences, University of Calgary, Calgary, AB Canada; 4 Department of Interventional Cardiology and Angiology, Institute of Cardiology, Warsaw, Poland; 5 Department of Nuclear Cardiology, Assuta Medical Centers, Tel Aviv, and Ben Gurion University of the Negev, Beer Sheba, Israel; 6 Department of Radiology, Division of Nuclear Medicine and Molecular Imaging, Brigham and Women’s Hospital, Boston, MA USA; 7 Division of Cardiology, Department of Medicine, and Department of Radiology, Columbia University Irving Medical Center and New York-Presbyterian Hospital, New York, NY USA; 8 Section of Cardiovascular Medicine, Department of Internal Medicine, Yale University School of Medicine, New Haven, CT USA; 9 Cardiovascular Imaging Technologies LLC, Kansas City, MO USA; 10 Oregon Heart and Vascular Institute, Sacred Heart Medical Center, Springfield, OR USA; 11 Division of Cardiology, University of Ottawa Heart Institute, Ottawa, ON Canada; 12 Department of Advanced Biomedical Sciences, University of Naples “Federico II”, Naples, Italy; 13 Department of Nuclear Cardiology, Oklahoma Heart Hospital, Oklahoma City, OK USA; 14 Department of Nuclear Medicine, Cardiac Imaging, University Hospital Zurich, Zurich, Switzerland

**Keywords:** Machine learning, SPECT myocardial perfusion, Coronary artery disease, Cluster analysis, Cardiovascular risk

## Abstract

**Purpose:**

Patients with known coronary artery disease (CAD) comprise a heterogenous population with varied clinical and imaging characteristics. Unsupervised machine learning can identify new risk phenotypes in an unbiased fashion. We use cluster analysis to risk-stratify patients with known CAD undergoing single-photon emission computed tomography (SPECT) myocardial perfusion imaging (MPI).

**Methods:**

From 37,298 patients in the REFINE SPECT registry, we identified 9221 patients with known coronary artery disease. Unsupervised machine learning was performed using clinical (23), acquisition (17), and image analysis (24) parameters from 4774 patients (internal cohort) and validated with 4447 patients (external cohort). Risk stratification for all-cause mortality was compared to stress total perfusion deficit (< 5%, 5–10%, ≥10%).

**Results:**

Three clusters were identified, with patients in Cluster 3 having a higher body mass index, more diabetes mellitus and hypertension, and less likely to be male, have dyslipidemia, or undergo exercise stress imaging (*p* < 0.001 for all). In the external cohort, during median follow-up of 2.6 [0.14, 3.3] years, all-cause mortality occurred in 312 patients (7%). Cluster analysis provided better risk stratification for all-cause mortality (Cluster 3: hazard ratio (HR) 5.9, 95% confidence interval (CI) 4.0, 8.6, *p* < 0.001; Cluster 2: HR 3.3, 95% CI 2.5, 4.5, *p* < 0.001; Cluster 1, reference) compared to stress total perfusion deficit (≥10%: HR 1.9, 95% CI 1.5, 2.5 *p* < 0.001; < 5%: reference).

**Conclusions:**

Our unsupervised cluster analysis in patients with known CAD undergoing SPECT MPI identified three distinct phenotypic clusters and predicted all-cause mortality better than ischemia alone.

**Supplementary Information:**

The online version contains supplementary material available at 10.1007/s00259-023-06218-z.

## Introduction

Patients with known coronary artery disease are a heterogenous population with varied clinical and imaging characteristics. Despite advances in contemporary medical, interventional, and surgical management, there remains a subgroup of patients with known cardiovascular disease who are at high risk of cardiac events and mortality [1]. Improved methods to characterize patients with known coronary artery disease who are at increased risk of cardiac events would enable more personalized, targeted management, and guide the use of new medical therapies.

Myocardial perfusion imaging (MPI) with single-photon emission computed tomography (SPECT) is an established technique to identify myocardial ischemia and risk-stratify patients [2]. Quantitative information from SPECT can provide valuable additional prognostic information over and above visual assessment alone [3]. Recently, the multi-center REgistry of Fast Myocardial Perfusion Imaging with NExt generation SPECT (REFINE SPECT) registry has been established, which aims to create a comprehensive clinical and imaging database of the latest generation SPECT images which are processed with quantitative software [4]. Supervised machine learning has been used to combine clinical and quantitative imaging features to improve prognostic assessment of patients undergoing SPECT [3, 5–7]. However, unsupervised machine learning has the potential to identify new cardiovascular phenotypes with unique prognostic implications.

Unsupervised machine learning aims to identify groups, or clusters, of patients which have similar combinations of characteristics, without the impact of biases from clinical experts or information on subsequent outcomes. Unsupervised learning differs from more commonly applied supervised methods by learning to separate data distributions into clusters, rather than being trained to predict specific classification or regression outcomes. This distinction allows unsupervised methods to unveil new patterns in cardiovascular diseases, to develop new understanding of disease phenotypes, and to identify novel high-risk groups without outcome bias. Cluster analysis has previously been used to identify clinical and imaging features that predict the risk of future cardiovascular events using magnetic resonance imaging, coronary computed tomography angiography, and echocardiography [8–10]. However, this technique has not previously been applied to MPI (SPECT or PET) or shown to improve prognostication for patients with known coronary artery disease in any imaging modality. Patients with known coronary artery disease represent a unique clinical challenge as a subset of these patients are at the highest risk of myocardial infarction, whereas others remain event free. Of the currently available prognostic scores for patients with known coronary artery disease, few incorporate these non-invasive imaging metrics, and their performance is low [11].

This study aims to use unsupervised machine learning to identify clusters amongst patients with known coronary artery disease who underwent SPECT MPI, and to assess how these phenotypic clusters differ in terms of all-cause mortality and subsequent cardiac events.

## Materials and methods

### Study design

In this multicenter, retrospective analysis of imaging and clinical data from the expanded REFINE SPECT registry [4], we performed unsupervised machine learning to identify phenotypic clusters amongst patients with known coronary artery disease who had undergone SPECT MPI, and to assess the association of these cluster groups with outcomes. The study complied with the Declaration of Helsinki and was approved by the institutional review boards of local sites and Cedars-Sinai Medical Center.

### Study population

**Fig. 1:**
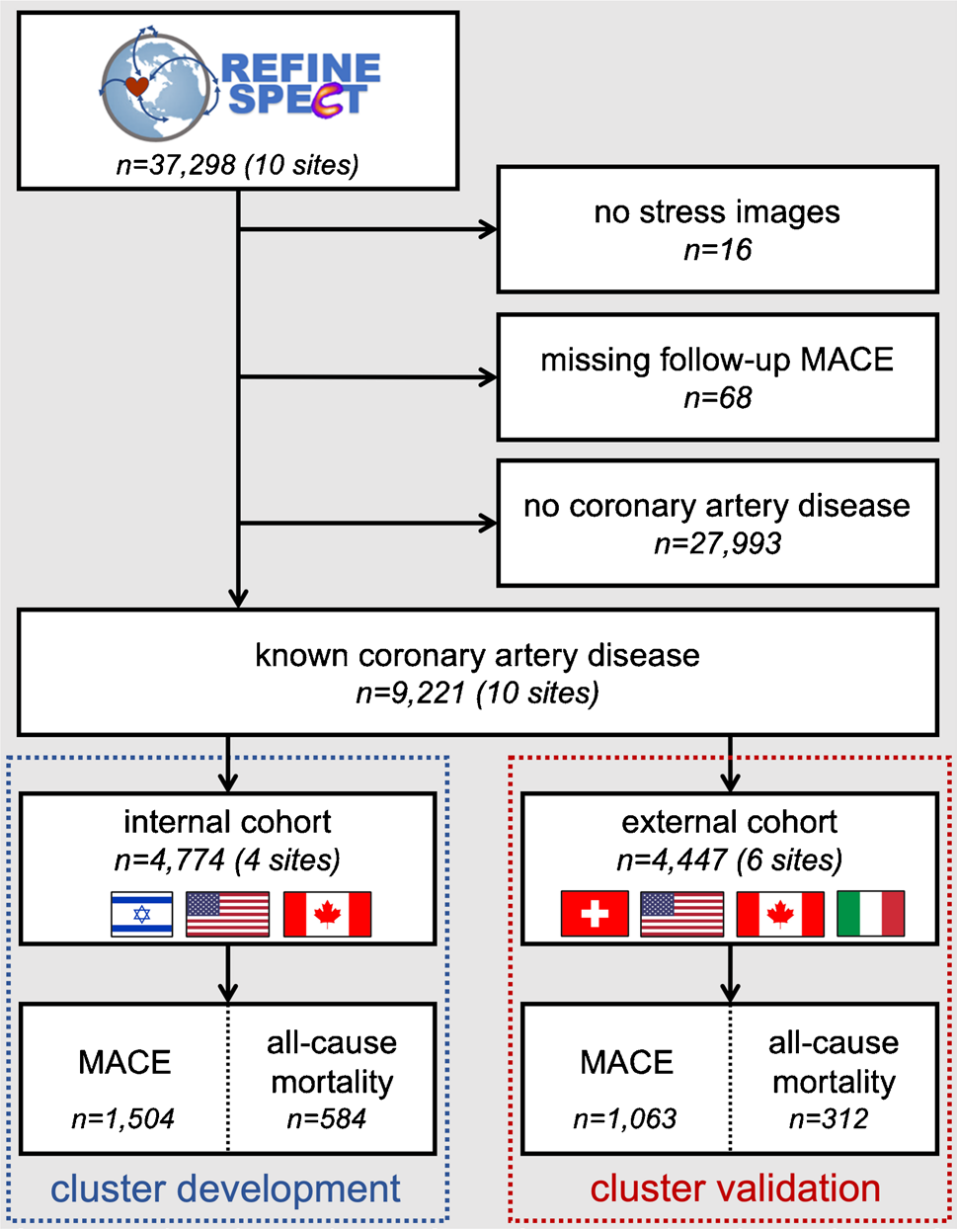
Consort diagram. Inclusion and exclusion criteria for retrospective analysis. MACE, major adverse cardiovascular event; REFINE SPECT, REgistry of Fast Myocardial Perfusion Imaging with NExt generation SPECT

The REFINE SPECT registry is an international multicenter registry of consecutive patients undergoing clinically indicated SPECT MPI which currently includes 37,298 patients from 10 worldwide sites [4]. From this registry, we selected patients with known coronary artery disease, defined as those with (one or more of) previous myocardial infarction, percutaneous coronary intervention, or coronary artery bypass grafting. Inclusion and exclusion criteria are detailed in Fig. 1. Patients were excluded if stress imaging was not available (*n* = 16), if follow-up information on death or major adverse cardiovascular events (MACE) was incomplete (*n* = 68), or if they had no previous history of coronary artery disease (*n* = 27,993).

### Clinical information

Clinical information was obtained from the REFINE SPECT registry database and included demographic information, cardiovascular risk factors, past medical history, and resting electrocardiogram (ECG) findings (Supplementary Table 1).

### SPECT MPI

SPECT MPI was performed at 10 sites (Assuta Medical Center, Tel Aviv, Israel; Brigham and Women’s Hospital, Boston, USA; Cedars-Sinai Medical Center, Los Angeles, CA, USA; Oklahoma Heart Hospital, Oklahoma City, OK, USA; Oregon Heart and Vascular Institute, Springfield, OR, USA; Ottawa Heart Institute, Ottawa, Ontario, Canada; University of Calgary, Calgary, Alberta, Canada; University of Naples, Naples, Italy; Yale University, New Haven, CT, USA; University Hospital Zurich, Zurich, Switzerland) with three different scanners (GE Discovery NM 530c, GE Discovery 570c, GE Healthcare, Haifa, Israel, and D-SPECT, Spectrum Dynamics, Haifa, Israel). SPECT acquisition parameters were recorded, including the type of stress performed, the physiological and clinical response to stress, and the isotope type and dose (Supplementary Table 1).

Quantitative SPECT MPI parameters were generated automatically using the Quantitative Perfusion SPECT (QPS)/Quantitative Gated SPECT (QGS) software (Cedars-Sinai Medical Center, Los Angeles, CA, USA) [12, 13]. Deidentified images were reviewed by core laboratory technologists, blinded to clinical data, for quality control. QPS/QGS was then used to generate myocardial contours and automatically generate quantitative SPECT MPI parameters including 24 stress/rest, gated/ungated, perfusion, and function parameters (parameters provided by group in Supplementary Table 1). Stress total perfusion deficit from a single position was classified as < 5%, 5–10%, or ≥10% for analysis. Quantitative percent ischemia was automatically determined as the difference between stress and rest total perfusion deficits, and was classified as < 5%, 5–10%, or ≥10% for analysis [3]. Single position acquisitions were obtained for 1,422/9,221 (15%) patients; therefore, we did not use two-position combined TPD.

### Data preprocessing

Data from four original REFINE SPECT sites (Assuta Medical Center, Brigham and Women’s Hospital, Cedars-Sinai Medical Center, Oregon Heart and Vascular Institute; *n* = 4774) were used as the internal cohort to perform unsupervised learning and to understand the clinical characteristics of the clusters. Data from six sites (Oklahoma Heart Center, Ottawa Heart Institute, University of Calgary, University of Naples, University Hospital Zurich, Yale University; *n* = 4447) was used as the external cohort to test impact of the cluster groups on outcomes (Fig. 1). All external site data was from the new REFINE SPECT sites, except for Ottawa, which was included with the external set to balance the sizes of the internal training and external testing cohorts. Clinical and imaging characteristics for internal and external cohorts are provided in Supplementary Table 2.

Data preprocessing to provide the machine learning algorithm with clean, uniform, and consistent data was performed. Machine learning analysis was performed in Python (version 3.9.7) using clinical information (23 parameters), acquisition parameters (17 parameters), and quantitative image analysis parameters (24 parameters; Supplementary Table 1). Visual SPECT-MPI assessments were not used to avoid potential clinician biases. Cardiovascular events and mortality were not used in the analysis because we wanted to develop a model that could phenotype and derive new pathophysiologic insights for patients at the time of imaging without bias towards specific outcomes. Unsupervised cluster analysis fits these requirements as the model learns to separate patients according to their individual data profile without a priori exposure to any outcome. Features with > 25% missingness were dropped from the set used for model fitting. Missing variables were imputed using median imputation for continuous variables and mode imputation for categorical variables. Data normalization was applied only when selected as an optional hyperparameter (Supplementary Table 3).

### Clinical outcomes

Patients were followed up for the occurrence of revascularization, myocardial infarction, unstable angina, percutaneous coronary intervention, coronary artery bypass grafting, and all-cause mortality. Major adverse cardiovascular events (MACE) were defined as coronary revascularization, myocardial infarction, admission for unstable angina, or all-cause mortality. Prognostic information for some of this population at 5 years has previously been reported [3].

### Unsupervised machine learning

Our primary tool is an unsupervised learning model that assigns patients to novel clusters for further analysis. This model first maps high dimensional patient data to a much lower-dimensional embedding space where patients can be efficiently clustered.

Dimensionality reduction was performed using the non-linear Uniform Manifold Approximation and Projection (UMAP) toolkit (UMAP Learn, version 0.5.2) [14]. Dimensionality reduction improves the performance of cluster analysis by simplifying the input feature space prior to clustering, reducing computation time and noise, while preserving the global data structure (i.e., the relative relationships between patients in the data) [14]. Traditional distance metrics break down at high dimensions, necessitating dimensionality reduction prior to clustering [15]. UMAP was selected as the primary engine for our unsupervised pipeline as it utilizes non-linear manifold approximation theory to estimate a low-dimensional data representation in a more efficient and scalable manner than other commonly used methods, while retaining a stable model representation that is saved and viable for clinical deployment [16]. Cao et al. demonstrated the robustness of UMAP to embed high dimensional data from cellular biology into a new representation, leading to fewer clusters than other commonly used methods [17]. The nature of our high-dimensional and multi-modal application supports the application of UMAP, which is expected to maintain performance as imaging technology advances and the total number of imaging variables grows. Reduction to three dimensions prior to clustering was selected to balance visualization of formed clusters with the embedding complexity. UMAP models were tested with classical clustering algorithms (hierarchical, *k*-means, gaussian mixture model; Scikit-Learn package, version 1.0.1) during internal model selection and validation.

A grid search was used to select the optimal dimensionality reduction parameters, clustering method, and number of clusters. Parameter ranges presented in Supplementary Table 3 were selected with the intention of producing a wide range of embedding structures and clustering combinations. Each set of parameters was evaluated and compared using the silhouette coefficient, with the optimal model being the configuration with the highest mean silhouette coefficient across the entire grid search. Silhouette coefficients are a standard metric to assess how well clusters are separated by assessing the separation distance of individuals between and within a cluster [18]. Silhouette scores (range: -1 to 1) increase as the distances to other patients in the same cluster decrease and the distances to patients from other clusters increase. They are similar to cluster-comparison metrics like the Davies-Bouldin and Calinski-Harabasz indices, with the advantage of providing in-built normalization so that scores are not biased to cluster sizes.

To validate the clinical utility of the unsupervised clustering, the model was tested in the external cohort. The trained dimensionality reduction model and the coordinates of the three cluster centroids derived in the internal cohort were used to assign external cohort patients to similar clusters.

### Statistical analysis

Statistical analysis was performed using R (version 4.1.1). Normally distributed data are presented with mean and standard deviation. Data that are not normally distributed are presented as median and interquartile range (IQR). Categorical data are presented as number and percentage. Statistical significance was assessed using Mann–Whitney Wilcoxon, Kruskal–Wallis rank sum, or Pearson’s chi-squared test. The hypergeometric distribution *v*-test (FactoMine R package, version 2.4) was performed to assess the representation of variables within each cluster, with a positive *v*-test score indicating over-representation of the variable within the cluster and a negative v-test score indicating under-representation [19]. Outcome data were analyzed using Cox proportional-hazards analysis with hazard ratios (HR), and 95% confidence intervals (CI) were calculated and compared using the global log-rank test and Wald test. Kaplan–Meier curves were constructed. A two-sided *p*-value < 0.05 was considered statistically significant. Separate survival analysis disaggregated by sex is presented according to the recommended use of SAGER guidelines for sex and gender equity in research [20].

## Results

### Study population

From 37,298 patients in the expanded REFINE SPECT registry, we identified 9221 patients with known coronary artery disease from ten sites where both clinical and imaging data was available. Cluster analysis was performed using data from 4774 patients in the internal cohort. These patients had a median age of 67 [IQR 60 to 75] years, and 78% (*n* = 3704) were male (Table 1). Forty-five percent (*n* = 2166) had a previous myocardial infarction, 71% (*n* = 3409) had previous percutaneous coronary intervention, and 2.5% (*n* = 121) had previous coronary artery bypass grafting. Similar demographic characteristics were present in the external cohort (Supplementary Table 4).

**Table 1:** Demographic characteristics for all patients and clusters in the internal cohort

		All patients	Cluster 1	Cluster 2	Cluster 3	*P*
*N*		4774	2005	1580	1189	-
Age (years)		67 (60, 75)	64 (57, 71)	70 (63, 77)	69 (61, 78)	** < 0.001**
Male		3704 (78%)	1667 (83%)	1264 (80%)	773 (65%)	** < 0.001**
BMI (kg/m^2^)		28 (25, 31)	27 (25, 30)	28 (25, 31)	29 (26, 33)	** < 0.001**
Hypertension		3658 (77%)	1480 (74%)	1166 (74%)	1012 (85%)	** < 0.001**
Diabetes mellitus		1736 (36%)	590 (29%)	646 (41%)	500 (42%)	** < 0.001**
Dyslipidemia		4067 (85%)	1766 (88%)	1342 (85%)	959 (81%)	** < 0.001**
Family history of CAD		1169 (24%)	629 (31%)	220 (14%)	320 (27%)	** < 0.001**
Smoking		594 (12%)	214 (11%)	202 (13%)	178 (15%)	**0.002**
Previous myocardial infarction		2166 (45%)	915 (46%)	677 (43%)	574 (48%)	**0.017**
Previous cardiac surgery or intervention	PCI	3409 (71%)	1540 (77%)	1143 (72%)	726 (61%)	**0.021**
	CABG	121 (2.5%)	53 (2.6%)	36 (2.3%)	32 (2.7%)	0.7
	TAVR	21 (0.4%)	3 (0.1%)	0 (0%)	18 (1.5%)	** < 0.001**
	Cardiac transplant	37 (0.8%)	1 (< 0.1%)	1 (< 0.1%)	35 (2.9%)	** < 0.001**
	Other	29 (0.6%)	3 (0.1%)	0 (0%)	26 (2.2%)	** < 0.001**
Presenting symptoms	Asymptomatic	2595 (54%)	1166 (58%)	904 (57%)	525 (44%)	** < 0.001**
	Atypical angina	993 (21%)	352 (18%)	232 (15%)	409 (34%)	
	Non-anginal	839 (18%)	363 (18%)	319 (20%)	157 (13%)	
	Typical	347 (7.3%)	124 (6.2%)	125 (7.9%)	98 (8.2%)	
Resting ECG abnormal		3526 (74%)	1416 (71%)	1222 (77%)	888 (75%)	** < 0.001**
Left ventricular hypertrophy		71 (1.5%)	30 (1.5%)	0 (0%)	41 (3.4%)	** < 0.001**

Number (%), median (interquartile range), bold indicates *p* < 0.05

*BMI* body mass index, *CABG* coronary artery bypass graft, *CAD* coronary artery disease, *ECG* electrocardiogram, *PCI* percutaneous coronary intervention, *PVD* peripheral vascular disease, *TAVR* transcutaneous aortic valve replacement

### Unsupervised machine learning

**Fig. 2:**
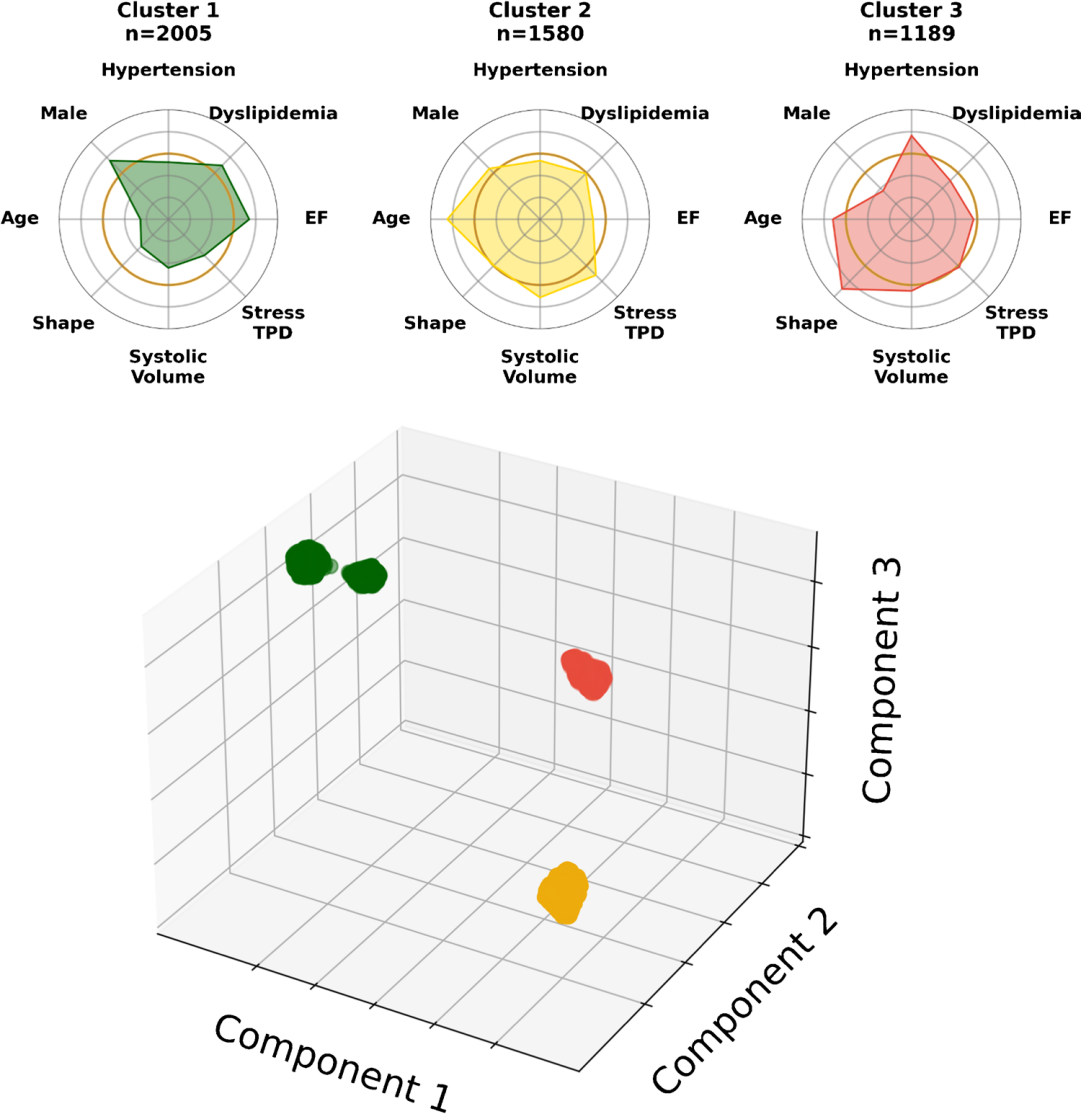
Distribution of clustered patients in the internal cohort. Top: radial plots provide a normalized summary of key clinical and quantitative imaging features in phenotypic clusters. Bottom: embedding of internal cohort patients in the reduced embedding space. Components of the embedding space are independent summary measures that combine multiple input parameters, which are determined by the non-linear dimensionality reduction process. Cluster 1: green, Cluster 2: yellow, Cluster 3: red. EF, stress ejection fraction; TPD, total perfusion deficit

The optimal unsupervised clustering method used the Braycurtis distance metric for dimensionality reduction with UMAP and *K*-means clustering. This model identified 3 optimal clusters, with a silhouette score of 0.93 (Supplementary Fig. 1). Based on clinical, acquisition, and quantitative imaging parameters, patients in the internal cohort were divided into three phenotypic clusters which were called Cluster 1 (*n* = 2005), Cluster 2 (*n* = 1580), and Cluster 3 (*n* = 1189; Fig. 2). This method was used to assign patients in the external cohort into three distinct clusters (Cluster 1, *n* = 1799; Cluster 2, *n* = 2213; Cluster 3, *n* = 435) which were used for assessment of cardiovascular outcomes.

### Clinical phenotypes of clusters

Patients in Cluster 3 had a higher body mass index and more hypertension, diabetes mellitus, smoking (*p* < 0.01 internal), and peripheral vascular disease (*p* < 0.001 for all others; Table 1; Supplementary Table 4). They were more likely to have had a previous myocardial infarction (*p* < 0.05 internal), to present with typical or atypical symptoms, and to have an abnormal resting electrocardiogram (*p* < 0.001 for all others). However, they were less likely to be male or have dyslipidemia (*p* < 0.001 for all). *V*-test scores showed that the clinical parameters which were the most over-represented in Cluster 3 were peripheral vascular disease, rest systolic blood pressure, symptoms of atypical angina, body mass index, and female gender (Supplementary Fig. 2).

In contrast, patients in Cluster 1 were younger, more likely to be male and have dyslipidemia, a family history of coronary artery disease, and be asymptomatic (*p* < 0.001 for all; Table 1; Supplementary Table 4). *V*-test scores showed that the clinical parameters which were over-represented in the Cluster 1 were height, family history of coronary artery disease, and male gender (Supplementary Fig. 2).

### Imaging phenotypes of clusters

Patients in Cluster 3 were less likely to undergo exercise stress or have abnormal electrocardiogram response to stress than patients in Cluster 1 (*p* < 0.001 for all; Table 2; Supplementary Table 4). *V*-test scores showed that pharmacological stress, stress administered activity, and ischemic or non-diagnostic clinical response to stress were over-represented in Cluster 3 (*p* < 0.001 for all; Supplementary Fig. 2). In contrast, patients in Cluster 1 were more likely to undergo exercise stress and have an abnormal electrocardiogram response to stress (*p* < 0.001 for all; Table 2; Supplementary Table 4). In Cluster 1, *v*-test scores showed that exercise stress, exercise duration, stress peak heart rate, positive heart rate response, and stress systolic blood were over-represented (*p* < 0.001 for all; Supplementary Fig. 2).

**Table 2:** SPECT acquisition characteristics for all patients and clusters in the internal cohort

		All patients	Cluster 1	Cluster 2	Cluster 3	*P*
*N*		4774	2005	1580	1189	-
Stress type	Exercise	1987 (42%)	1986 (99%)	0 (0%)	1 (< 0.1%)	** < 0.001**
	Pharmacological	2787 (58%)	19 (1%)	1580 (100%)	1188 (> 99%)	
Clinical response to stress	Non-ischemic	3196 (76%)	1518 (80%)	1302 (90%)	376 (43%)	** < 0.001**
	Equivocal	187 (4.4%)	53 (2.8%)	0 (0%)	134 (15%)	
	Ischemic or abnormal	842 (17.6%)	332 (16.6%)	148 (9.3%)	362 (30.4%)	
ECG response to stress	Negative	2552 (54%)	1068 (53%)	790 (50%)	694 (58%)	** < 0.001**
	Borderline or equivocal	335 (7%)	222 (11.1%)	84 (5.3%)	29 (2.4%)	
	Positive	513 (10.7%)	370 (18.4%)	96 (6.1%)	47 (4.0%)	
Heart rate (Beats per minute)	Rest	68 [60, 77]	69 [60, 79]	67 [60, 76]	67 [59, 76]	** < 0.001**
	Stress peak	110 [88, 137]	141 [131, 150]	92 [80, 105]	90 [78, 104]	** < 0.001**
Systolic blood pressure (mmHg)	Rest	130 [120, 140]	130 [120, 140]	130 [120, 140]	140 [124, 158]	** < 0.001**
	Stress peak	150 [130, 170]	168 [154, 180]	140 [120, 150]	131 [119, 150]	** < 0.001**
Diastolic blood pressure (mmHg)	Rest	80 [74, 80]	80 [78, 80]	80 [80, 80]	76 [68, 83]	** < 0.001**
	Stress peak	80 [70, 80]	80 [80, 80]	80 [80, 80]	70 [62, 80]	** < 0.001**
Location	Inpatient	444 (9.3%)	115 (5.7%)	0 (0%)	329 (28%)	** < 0.001**
	Outpatient	4285 (90%)	1871 (93%)	1580 (100%)	834 (70%)	
	Emergency	43 (0.9%)	19 (0.9%)	0 (0%)	24 (2.0%)	
Dose (MBq)	Rest	555 (318, 925)	555 (303, 925)	925 (666, 925)	316 (285, 352)	** < 0.001**
	Stress	370 (259, 973)	370 (259, 688)	333 (222, 370)	1214 (888, 1363)	** < 0.001**

Number (%), median (interquartile range), bold indicates *p* < 0.05

*ECG* electrocardiogram

**Fig. 3:**
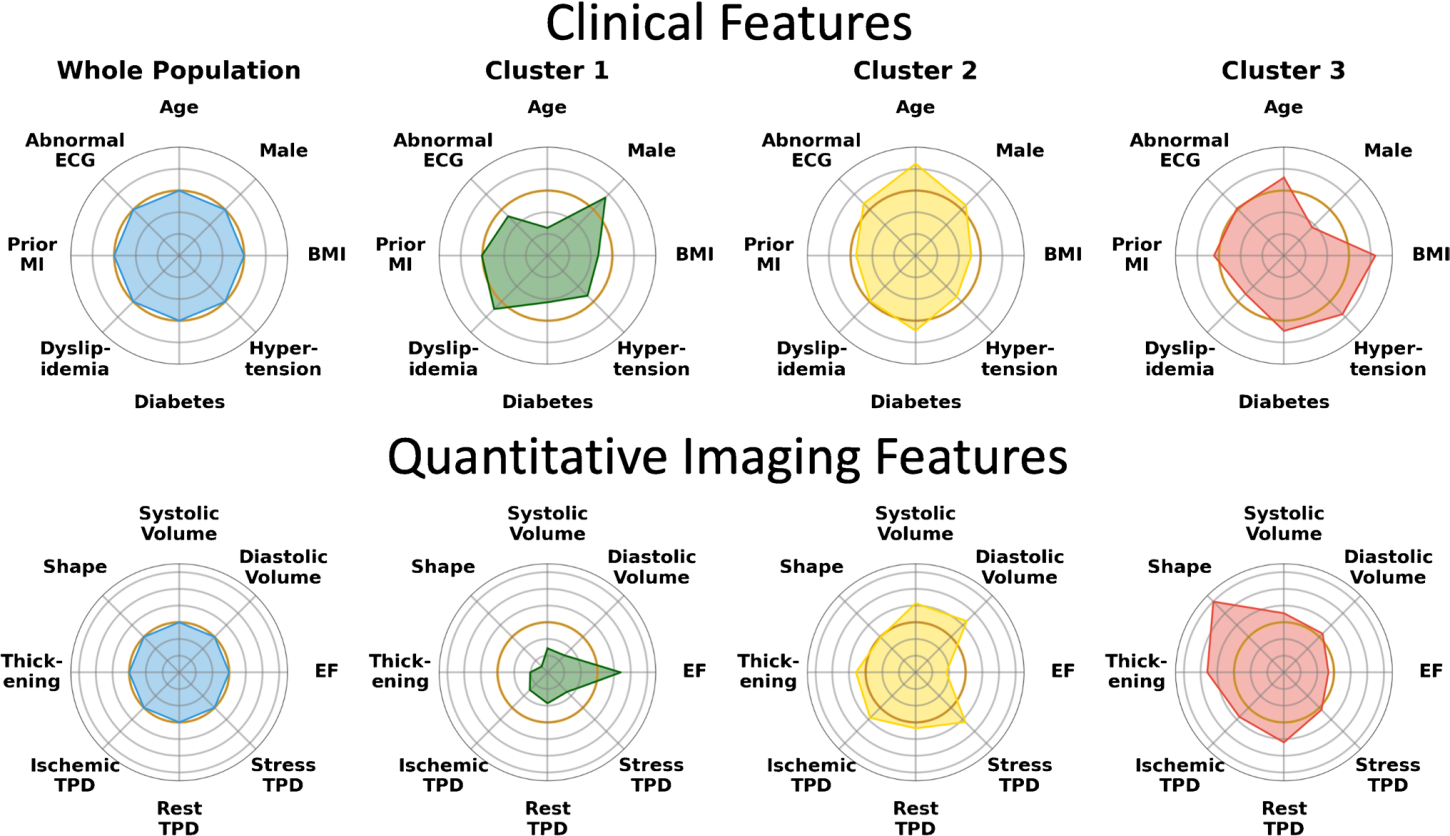
Clinical and quantitative imaging features in phenotypic clusters. Radial plots show differences in the pattern of clinical features and quantitative imaging features in the internal cohort, with the whole population (blue), Cluster 1 (green), Cluster 2 (yellow), and Cluster 3 (red). The orange ring represents the whole population; inside this ring demonstrates under-representation and outside this ring demonstrates over-representation. ECG, electrocardiogram; EF, stress ejection fraction; MI, myocardial infarction; TPD, total perfusion deficit

Quantitative SPECT-MPI analysis showed that patients in Cluster 3 had higher rest total perfusion deficit, and stress gated shape index on end diastolic and end systolic images (Fig. 3; Supplementary Table 5). Patients in Cluster 2 had the highest stress total perfusion deficit, percent ischemia, and stress and rest gated end diastolic and systolic volumes (*p* < 0.001 for all). Patients in Cluster 1 had the highest stress and rest ejection fraction (Supplementary Table 5).

### Cardiovascular outcomes

During a median follow-up of 4.2 [3.3, 5.1] years, all-cause mortality occurred in 584 patients (12%) and MACE in 1504 (32%) patients in the internal cohort (Table 3). Patients in Cluster 3 in the internal cohort were more likely to experience myocardial infarction, unstable angina, early revascularization, MACE, and all-cause mortality, but not all revascularization or coronary artery bypass grafting (Table 3).

**Table 3:** Cardiovascular outcomes for all patients and clusters in the internal cohort

	All	Unsupervised machine learning				Stress total perfusion deficit			
		Cluster 1	Cluster 2	Cluster 3	*P*	< 5%	5–10%	≥10%	*P*
N	4774	2005	1580	1189	-	2394	958	1422	-
Early revascularization*	258 (5.4%)	87 (4.3%)	107 (6.8%)	64 (5.4%)	**0.006**	40 (1.7%)	49 (5.1%)	169 (12%)	** < 0.001**
All revascularization	922 (19%)	369 (18%)	336 (21%)	217 (18%)	0.055	359 (15%)	191 (20%)	372 (26%)	** < 0.001**
PCI	827 (17%)	326 (16%)	308 (19%)	193 (16%)	**0.021**	315 (13%)	181 (19%)	331 (23%)	** < 0.001**
CABG	121 (2.5%)	53 (2.6%)	36 (2.3%)	32 (2.7%)	0.7	50 (2.1%)	18 (1.9%)	53 (3.7%)	**0.003**
MI	156 (3.3%)	40 (2.0%)	61 (3.9%)	55 (4.6%)	** < 0.001**	62 (2.6%)	33 (3.4%)	61 (4.3%)	**0.016**
Unstable angina	157 (3.3%)	62 (3.1%)	1 (< 0.1%)	94 (7.9%)	** < 0.001**	77 (3.2%)	23 (2.4%)	57 (4.0%)	0.094
MACE	1504 (32%)	494 (25%)	513 (32%)	497 (42%)	** < 0.001**	584 (24%)	320 (33%)	600 (42%)	** < 0.001**
All-cause mortality	584 (12%)	97 (4.8%)	193 (12%)	294 (25%)	** < 0.001**	201 (8.4%)	129 (13%)	254 (18%)	** < 0.001**

Number (%), bold indicates *p* < 0.05

*CABG* coronary artery bypass graft, *MACE* major adverse cardiovascular events, *PCI* percutaneous coronary intervention

^*^90 days

During a median follow-up of 2.6 [0.14, 3.3] years, all-cause mortality occurred in 312 (7%) of patients and MACE occurred in 1063 (24%) patients in the external cohort (Supplementary Table 5). Patients in Cluster 3 in the external cohort were more likely to experience myocardial infarction, unstable angina, MACE, all-cause mortality, and revascularization, but not coronary artery bypass grafting (Supplementary Table 6).

**Fig. 4:**
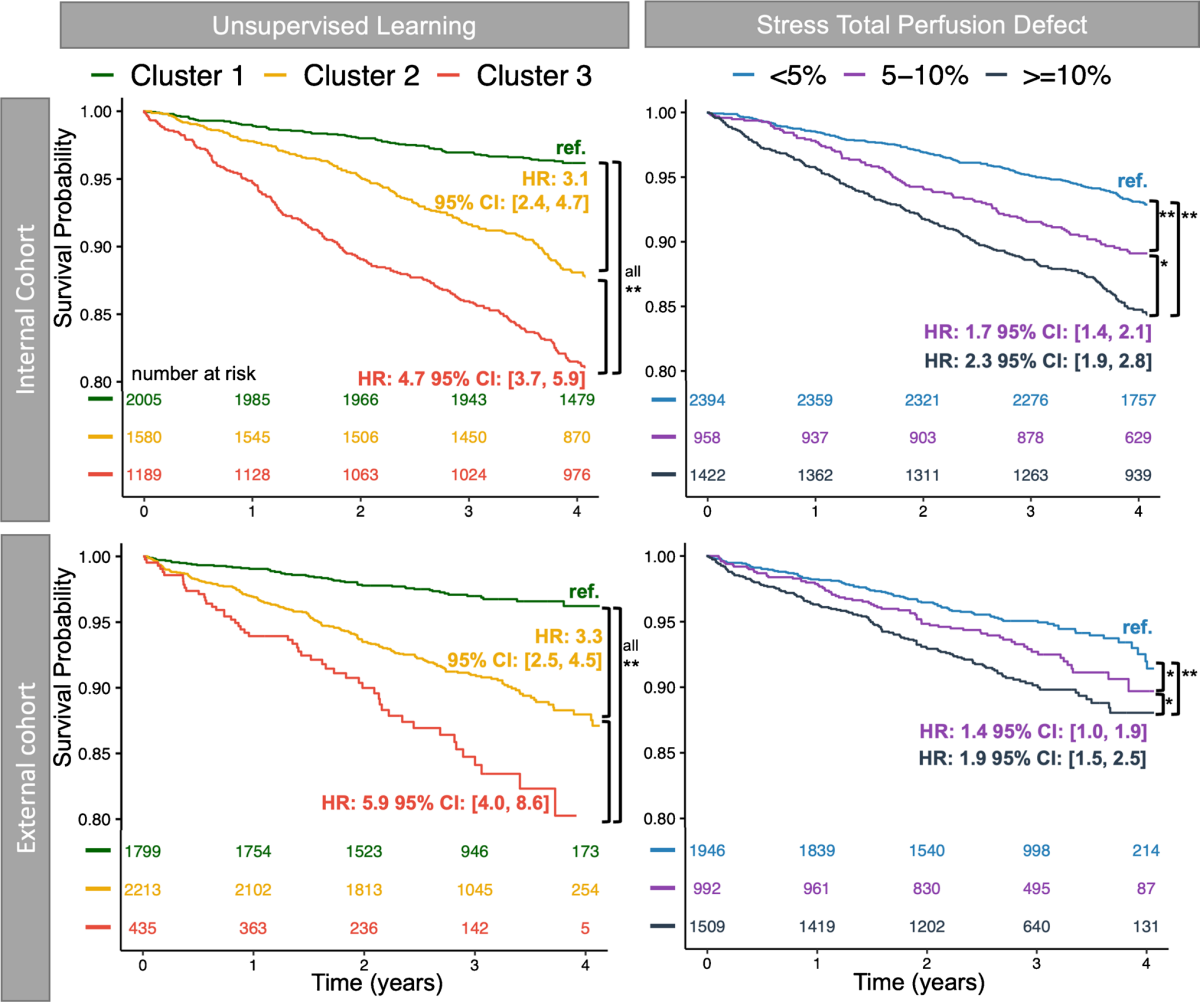
Clinical implications of phenotypic clustering in terms of all-cause mortality. Kaplan–Meier curves for all-cause mortality by unsupervised learning clusters demonstrate strong risk stratification compared to stress total perfusion deficit in internal and external cohorts. ** indicates *p* < 0.001; * indicates *p* < 0.05

In the external cohort, all-cause mortality was almost six times more likely in Cluster 3 compared to Cluster 1 (HR 5.9, 95% CI 4.0 to 8.6, *p* < 0.001) and three times more likely in Cluster 2 (HR 3.3, 95% CI 2.5 to 4.5, *p* < 0.001; Fig. 4). In contrast, stress total perfusion deficit provided less risk differentiation between groups for all-cause mortality (HR 1.9, 95% CI 1.5 to 2.5, *p* < 0.001 for stress total perfusion deficit ≥10% versus < 5%; Fig. 4). Similarly, ischemia provided less differentiation between groups for all-cause mortality (HR 1.8, 95% CI 1.3 to 2.5, *p* < 0.001 for quantitative percent ischemia ≥10% versus < 5%; Supplementary Fig. 3).

**Fig. 5:**
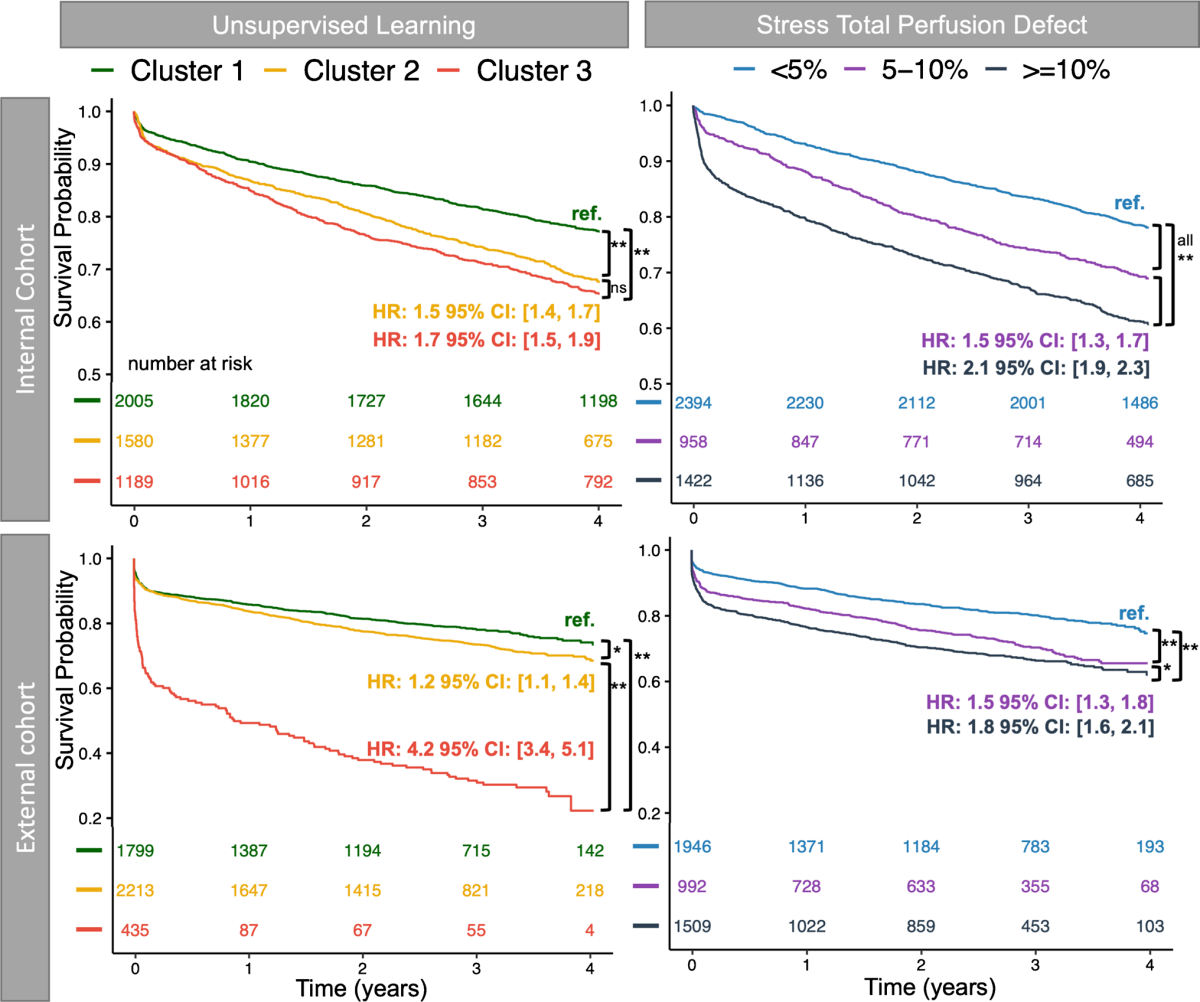
Clinical implications of phenotypic clustering in terms of MACE. Kaplan–Meier curves for MACE by unsupervised learning clusters demonstrate strong risk stratification compared to stress total perfusion deficit in internal and external cohorts. ** indicates *p* < 0.001; * indicates *p* < 0.05; ns indicates *p* ≥ 0.05

In the external cohort, MACE was also more likely to occur in Cluster 3 (HR 4.2, 95% CI 3.4 to 5.1, *p* < 0.001), and Cluster 2 (HR 1.2, 95% CI 1.1 to 1.4, *p* = 0.002), compared to Cluster 1. In contrast, stress total perfusion deficit (HR 1.8, 95% CI 1.6 to 2.1, *p* < 0.001 for stress total perfusion deficit ≥10% versus < 5%) and ischemia (HR 2.1, 95% CI 1.7 to 2.4, *p* < 0.001 for quantitative percent ischemia ≥10% versus < 5%) provided less differentiation between groups for MACE (Fig. 5).

Results of Cox proportional hazards analysis for clusters disaggregated according to sex for both internal and external cohorts. Supplementary Tables 7–10 demonstrate consistent risk-stratification of male and female patients for all-cause mortality and MACE outcomes by learned clusters. Supplementary Tables 11–12 demonstrate that risk-stratification by site in the training population is inferior to stratification by unsupervised clusters.

## Discussion

We have implemented an unsupervised machine learning approach to identify new phenotypic clusters amongst patients with known coronary artery disease undergoing SPECT MPI. Patients with known coronary artery disease are an understudied and heterogenous group, and improved risk stratification for this population will advance targeted management strategies. In this large multicenter registry, we have identified three clusters. These clusters demonstrated important differences in all-cause mortality and MACE, even though no outcome endpoints were included in the unsupervised machine learning model. The clustering was an unbiased approach based on clinical features, acquisition features, and fully automated quantitative image analysis features, without using information on cardiovascular events. The cluster assignment provided improved risk assessment compared to quantitative SPECT MPI ischemia alone. Importantly, we demonstrated excellent performance of the clustering approach in an external population not used for model training. In clinical practice, the use of these clusters could improve personalized management of coronary artery disease by robust identification of patients at low, medium, and high risks of all-cause mortality and MACE after SPECT MPI.

For patients with known coronary artery disease with stable chest pain despite optimal guideline directed medical therapy, stress imaging has a Class I indication in the current ACC/AHA guidelines [2]. Assessing the severity of ischemia can be used to guide decisions regarding the use and intensification of anti-anginal medications and the use of invasive coronary angiography [2]. In our study, we have shown that the clustering based on clinical, acquisition, and automated image analysis parameters can provide better stratification of risk compared to stress total perfusion deficit alone. This machine learning model was developed using automated SPECT MPI analysis, along with acquisition and clinical parameters, to provide an objective and reproducible input that is not dependent on the site experience and reading style. The cluster assignment can be available to clinicians at the time of reporting as an aid in the overall patient assessment.

Cluster analysis can also provide important information on the demographic characteristics of patients within each group, which can help to develop a new understanding of disease. In our study, the low-risk cluster was predominantly comprised of patients with established cardiovascular risk factors, but with overall good cardiovascular condition such as the ability to perform exercise stress and normal ejection fraction. In contrast, Cluster 3 represented patients with established cardiovascular risk factors but with poor cardiovascular condition, established findings of perfusion defects, and inability to perform exercise stress. We note that Cluster 3 has higher proportion of female patients compared to Clusters 1 and 2. While the reason for this is not clear, it may be that the unsupervised machine learning model is identifying combinations of risk factors and SPECT MPI findings that are more common in females. To our knowledge, this finding was not previously reported in the literature. Consistent risk stratification when results were disaggregated according to sex demonstrated that the dataset’s sex imbalance did not limit the applicability of this model to the minority group of female patients. Some variance between cluster characteristics could be explained by site-specific protocols; however, the improved risk stratification provided by the clusters compared to stratification by site in the internal training population suggests that the algorithm is identifying more than site alone. Thus, the combination of the clinical, acquisition, and quantitative image analysis findings using unsupervised machine learning can identify new groups of patients with known coronary artery disease. Such unsupervised machine learning (not directly trained on outcome data as in standard statistical and machine learning approaches) is robust and resistant to overfitting as demonstrated in our external testing of the clustering.

Automated quantitative analysis of SPECT MPI can provide additional information to improve risk stratification compared to visual assessment alone in a variety of sub-groups within the REFINE SPECT registry [3, 21, 22]. Quantitative assessment of changes in ventricular morphology such as shape and eccentricity indices has been shown to be independently associated with MACE [23]. Transient ischemic dilation and wall motion abnormalities have also been shown to identify patients with mild ischemia who are at increased risk [24]. There is an increasing number of clinical and imaging parameters which clinicians must synthesize in decision making regarding patient care. The cluster analysis provided in this paper provides a new method of synthesizing this information in an unbiased method-not directly driven by outcomes, to identify new and important phenotypic clusters. We have also shown in this paper that these phenotypic clusters have prognostic implications, which are robust in an external validation cohort. The trained models produced in this paper could be incorporated into semi-automatic SPECT software to automatically provide this information to physicians, but further work is required to assess the impact of this on decision making and patient care.

Supervised machine learning assessment of SPECT MPI parameters has previously been shown to be a better predictor of early coronary revascularization than assessment by a nuclear cardiologist or automatically quantified tissue perfusion defects [7]. In supervised machine learning, the computer model is trained based on knowledge of a defined outcome, such as mortality or MACE. In contrast, in unsupervised machine learning, the computational model is not provided with an outcome and instead seeks to understand patterns in the data and create clusters based on phenotypic similarities. Unsupervised machine learning has recently emerged as a useful technique to identify new phenotype-based groupings in complex diseases without the pre-conceived biases of existing categories. It has been used to identify new classifications in patients undergoing a variety of imaging tests, including patients with bicuspid aortopathy undergoing computed tomography [25], healthy volunteers in the UK Biobank study undergoing cardiac MRI [26], patients with left ventricular assist devices undergoing echocardiography preoperatively [27], and patients with aortic stenosis undergoing echocardiography [28]. It has also been used to identify new subtypes of patients based on clinical characteristics in a variety of diseases including heart failure [29], hypertension [30], type 2 diabetes mellitus [31], and amyloidosis [32]. Our paper represents the first time that unsupervised machine learning has been applied to SPECT MPI to improve clinical prognostication and the first time the output of unsupervised machine learning has been tested independently in an external population. Our unsupervised technique provides unbiased clustering of patients, not influenced by prior knowledge of the disease in question, as the model is trained without information on outcomes.

## Study limitations

We must acknowledge some limitations of our study. This was a large study with data from four sites used to create the unsupervised machine learning model and data from six sites used for external validation. However, it was a retrospective study, with heterogeneity in the imaging technique between sites, including referrals, imaging protocols, and administered radiotracer doses. The heterogeneity in patient populations does increase the generalizability of our findings. In addition, 78% of the patients included in this study were male, likely representing the pattern of disease in this population. Further work is therefore required with datasets from a larger number of centers including more women and using different protocols. Information on race and ethnicity is not available in the REFINE SPECT registry; therefore, further work is required to assess the impact of this machine learning tool in more diverse populations. Other methods to perform unsupervised machine learning which were not explored in this paper may have revealed different results. Additionally, the UMAP model does not provide interpretability along axis of the fit dimensions. However, our use of well-known quantitative parameters allows for detailed analysis and interpretability of the divisions into clusters according to clinical practice.

## Conclusion

In this study, unsupervised learning has identified new phenotypic clusters of SPECT MPI patients with known coronary artery disease. Despite not using outcomes during training, the model shows improved prognostic assessment as compared to standard quantitative measures. These clusters could be used to help clinicians in robust identification of high-risk patients and more personalized, targeted management.

## Supplementary Information

Below is the link to the electronic supplementary material.

Supplementary file1 (PDF 868 KB)

## Data Availability

The data underlying this article cannot be shared publicly due to the multi-institutional data sharing agreement and institutional review board (IRB) constraints. To the extent allowed by the data sharing agreements and IRB protocols, the data from this manuscript will be shared upon written and reasonable request to the corresponding author.

## Abbreviations

CAD: Coronary artery disease
CCTA: Coronary computed tomography angiography
MACE: Major adverse cardiac events
MPI: Myocardial perfusion imaging
MRI: Magnetic resonance imaging
REFINE SPECT: REgistry of Fast Myocardial Perfusion Imaging with NExt generation SPECT
SPECT: Single-photon emission computed tomography
