# Supplementary material for: Unsupervised learning to characterize patients with known coronary artery disease undergoing myocardial perfusion imaging
Source: Eur J Nucl Med Mol Imaging. 2023 Apr 17;50(9):2656–68. doi: 10.1007/s00259-023-06218-z (PMC10317876; doi:10.1007/s00259-023-06218-z)
Supplement: Supplementary file 1 — Supplementary file1 (PDF 868 KB) [file 259_2023_6218_MOESM1_ESM.pdf]

Supplemental Material

European Journal of Nuclear Medicine and Molecular Imaging

Unsupervised learning to characterize patients with known coronary artery disease undergoing myocardial perfusion imaging

Brief title: Cluster analysis in known coronary disease

Michelle C Williams MBChB PhD[A,B*], Bryan P Bednarski MSc[A*], Konrad Pieszko MD PhD[A], Robert JH Miller MD[A,C], Jacek Kwiecinski MD PhD[A,D], Aakash Shanbhag MSc[A], Joanna X Liang BA[A], Cathleen Huang BA[A], Tali Sharir MD[E], Sharmila Dorbala MD MPH[F], Marcelo F Di Carli MD[F], Andrew J Einstein MD PhD[G], Albert J Sinusas MD[H], Edward J Miller MD PhD[H], Timothy M Bateman MD[I], Mathews B. Fish MD[J], Terrence D. Ruddy MD[K], Wanda Acampa MD[L], M. Timothy Hauser MD[M], Philipp A Kaufmann MD[N], Damini Dey PhD[A], Daniel S. Berman MD[A], Piotr J. Slomka PhD[A]

* - authors contributed equally

A. Departments of Medicine (Division of Artificial Intelligence in Medicine), Biomedical Sciences, and Imaging, Cedars-Sinai Medical Center, Los Angeles, California, United States
B. British Heart Foundation Centre for Cardiovascular Science, University of Edinburgh, Edinburgh, United Kingdom
C. Department of Cardiac Sciences, University of Calgary, Calgary, Alberta, Canada
D. Department of Interventional Cardiology and Angiology, Institute of Cardiology, Warsaw, Poland
E. Department of Nuclear Cardiology, Assuta Medical Centers, Tel Aviv, and Ben Gurion University of the Negev, Beer Sheba, Israel
F. Department of Radiology, Division of Nuclear Medicine and Molecular Imaging, Brigham and Women's Hospital, Boston, Massachusetts, United States
G. Division of Cardiology, Department of Medicine, and Department of Radiology, Columbia University Irving Medical Center and New York-Presbyterian Hospital, New York, New York, United States
H. Section of Cardiovascular Medicine, Department of Internal Medicine, Yale University School of Medicine, New Haven, Connecticut, United States
I. Cardiovascular Imaging Technologies LLC, Kansas City, Missouri, United States
J. Oregon Heart and Vascular Institute, Sacred Heart Medical Center, Springfield, Oregon, United States
K. Division of Cardiology, University of Ottawa Heart Institute, Ottawa, Ontario, Canada
L. Department of Advanced Biomedical Sciences, University of Naples "Federico II", Naples, Italy
M. Department of Nuclear Cardiology, Oklahoma Heart Hospital, Oklahoma City, Oklahoma, United States
N. Department of Nuclear Medicine, Cardiac Imaging, University Hospital Zurich, Zurich, Switzerland

Address for correspondence

Piotr Slomka, PhD, FACC, FASNC, FCCPM, Cedars-Sinai Medical Center, 8700 Beverly Boulevard, Ste. Metro 203, Los Angeles, California 90048, Phone: 310-423-4348, piotr.slomka@cshs.org, Twitter handle: @Piotr_JSlomka

Table of Contents

Supplementary Figure 1 3
Supplementary Figure 2 4
Supplementary Figure 3 5
Supplementary Table 1 6
Supplementary Table 2 7
Supplementary Table 3 9
Supplementary Table 4 10
Supplementary Table 5 12
Supplementary Table 6 13
Supplementary Table 7 14
Supplementary Table 8 14
Supplementary Table 9 15
Supplementary Table 10 15
Supplementary Table 11 16
Supplementary Table 12 16

Supplementary Figure 1: Silhouette scores for varying number of clusters in the internal cohort. The optimal number of clusters, with the highest Silhouette score, was three clusters.

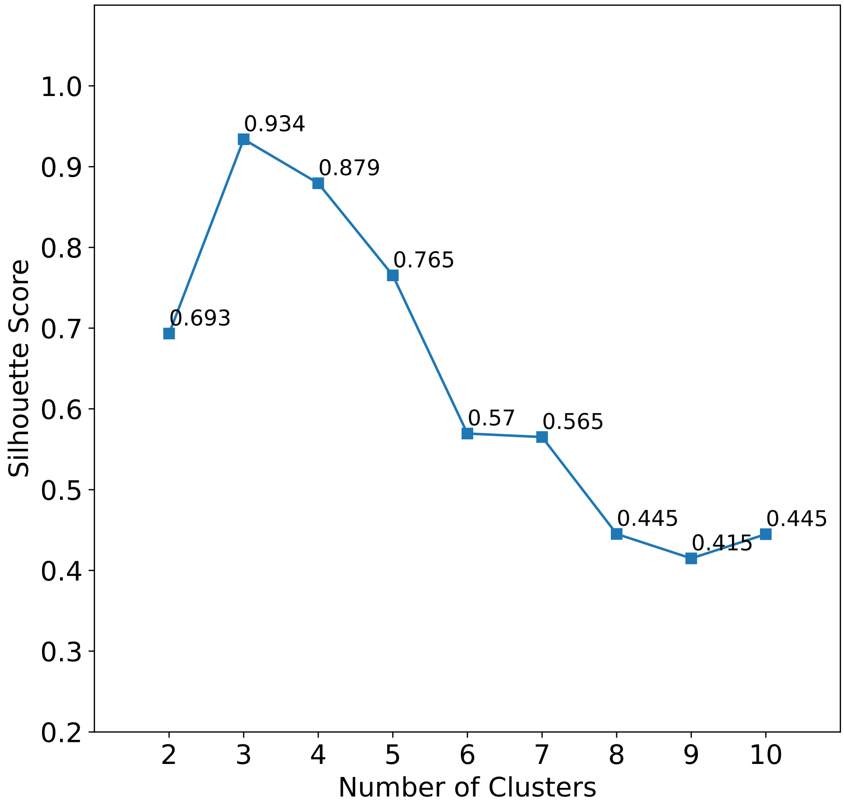

Supplementary Figure 2: Under- and over-representation of clinical (green), acquisition (orange) and image analysis (purple) features in Cluster 1, Cluster 2, and Cluster 3 in the internal cohort. All variables from the REFINE SPECT registry are analyzed here for their broad clinical interpretation, while only a subset of these were used to develop the model. Continuous variables are represented by a single entry, while categorical variables are encoded for each individual category. A positive v-test score indicates over-representation, and a negative v-test score indicates under-representation. Positive v-test scores for continuous variables (such as 'age') correspond to larger values on average for that cluster. All values shown possess a p<1e-8, to show only the most significant variables while maintaining figure interpretability.

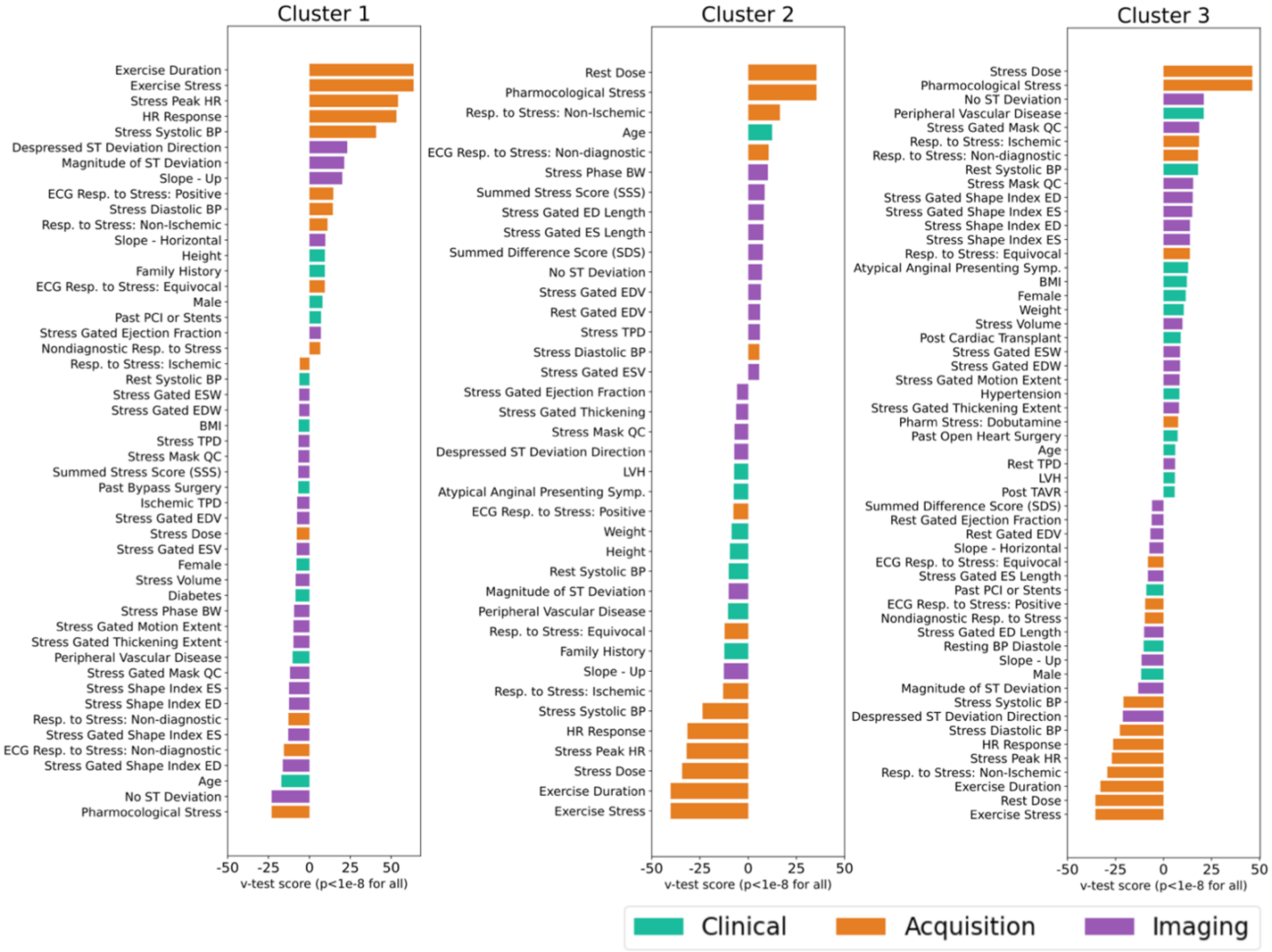

BMI, body mass index; BW, bandwidth; ECG, electrocardiogram; ED, end diastolic; EDW, end diastolic width; ES, end systolic; ESW end systolic width, EDV, end diastolic volume; ESV, end systolic volume; PCI, percutaneous coronary intervention; QC, quality control; TAVR, tricuspid aortic valve replacement, TPD, total perfusion defect.; LVH, left ventricular hypertrophy.

Supplementary Figure 3: Kaplan-Meier curves for all-cause mortality by unsupervised learning clusters demonstrate strong risk stratification compared to quantitative percent ischemia in internal and external cohorts.

** indicates p<0.001; * indicates p<0.05; ns indicates p>=0.05; TPD, total perfusion defect.

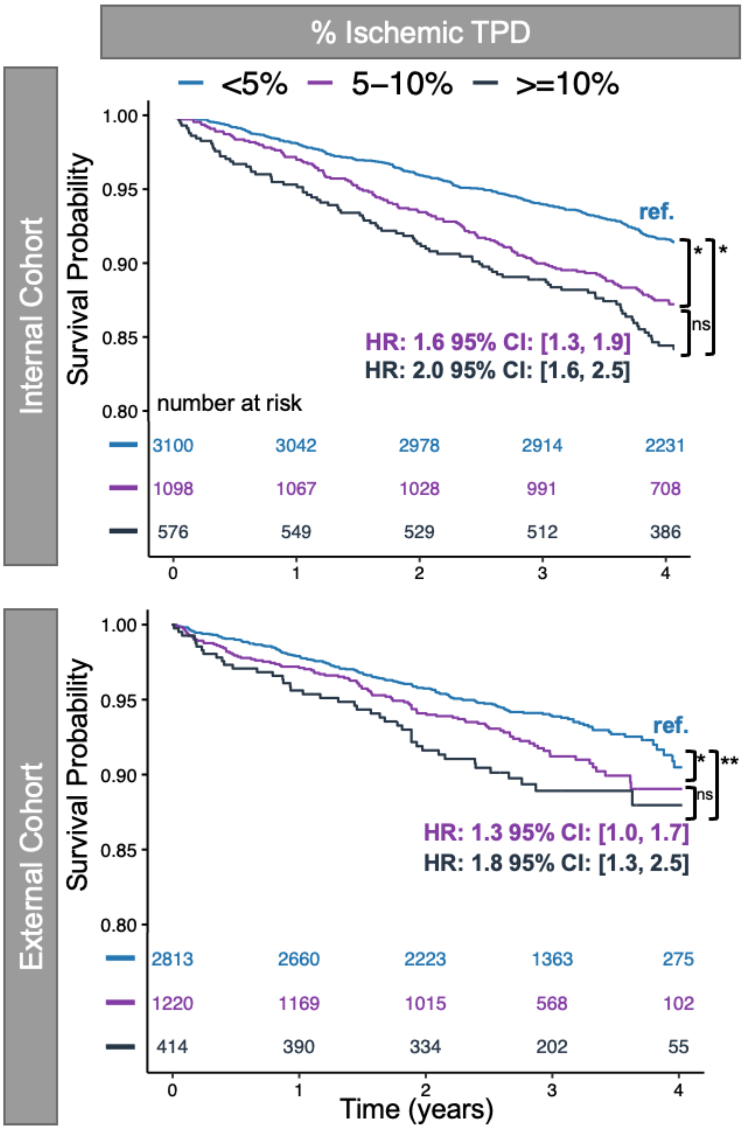

Supplementary Table 1: Parameters used for unsupervised cluster analysis.

Feature Group	Feature Names
Clinical parameters<br>(n=23)	Age<br>Gender<br>Height<br>Weight<br>Body Mass Index<br>Hypertension<br>Diabetes Mellitus<br>Dyslipidemia<br>Family History of Coronary Artery Disease<br>Smoking<br>Anginal Presenting Symptoms<br>Resting Heart Rate<br>Resting Blood Pressure Systole<br>Resting Blood Pressure Diastole<br>Rest ECG<br>Left Ventricular Hypertrophy<br>Past Myocardial Infarction<br>Peripheral Vascular Disease<br>Previous PCI or Stents<br>Previous TAVR<br>Previous Coronary Artery Bypass Surgery<br>Post Cardiac Transplant<br>Past Other Open-Heart Surgery
Acquisition parameters<br>(n=17)	Pharmacological or Exercise Stress<br>Pharmacological Stress Agent<br>Exercise Protocol<br>Peak Stress Heart Rate<br>Peak Stress Systolic Blood Pressure<br>Peak Stress Diastolic Blood Pressure<br>Exercise Duration<br>Heart Rate Response<br>ECG Response to Stress<br>Clinical Response to stress<br>Rest Dose<br>Rest Isotope<br>Stress Dose<br>Imaging Protocols<br>Magnitude of ST Deviation<br>ST Deviation Direction<br>Stress-Imaging Position
Quantitative imaging analysis parameters<br>(n=24)	Stress Total Perfusion Deficit<br>Stress Total Perfusion Deficit (combined 2 views)<br>Stress End Diastolic Volume<br>Stress End Diastolic Wall Volume<br>Stress End Diastolic Length<br>Stress Shape Index End Diastolic<br>Stress End Systolic Volume<br>Stress End Systolic Wall Volume<br>Stress End Systolic Length<br>Stress Shape Index End Systolic<br>Stress Ejection Fraction<br>Stress Thickening Abnormal Extent<br>Stress Average Thickening<br>Stress Motion Abnormal Extent<br>Stress Average Motion<br>Stress Volume<br>Stress Phase Bandwidth<br>Stress Perfusion Image Quality Control Flag<br>Stress Gated Image Quality Control Flag<br>Rest Total Perfusion Deficit<br>Rest End Diastolic Volume<br>Rest End Systolic Volume<br>Rest Ejection Fraction<br>Percent Ischemia

Supplementary Table 2: Demographic and imaging characteristics for all patients in the internal and external cohorts.

		All patients	Internal Cohort	External Cohort	P
N		9,221	4,774	4,447	-
Age (years)		68 (60, 75)	67 (60, 75)	68 (60, 76)	**<0.001**
Male		7,049 (76%)	3,704 (78%)	3,345 (75%)	**0.008**
BMI (kg/m^2)		27.7 (24.9, 31.0)	27.7 (25.1, 31.1)	27.5 (24.8, 30.9)	**0.010**
Hypertension		7,404 (80%)	3,658 (77%)	3,746 (84%)	**<0.001**
Diabetes Mellitus		3,266 (35%)	1,736 (36%)	1,530 (34%)	0.052
Dyslipidemia		6,952 (76%)	4,067 (85%)	2,885 (65%)	**<0.001**
Family History of CAD		3,098 (34%)	1,169 (24%)	1,929 (43%)	**<0.001**
Smoking		2,543 (28%)	594 (12%)	1,949 (44%)	**<0.001**
Previous MI		4,646 (50%)	2,166 (45%)	2,480 (56%)	**<0.001**
Previous Cardiac Surgery or Intervention	PCI	6,106 (66%)	3,409 (71%)	2,697 (61%)	**<0.001**
	CABG	2,720 (29%)	1,427 (30%)	1,293 (29%)	0.4
	TAVR	24 (0.3%)	21 (0.4%)	3 (<0.1%)	**<0.001**
	Other	62 (0.7%)	29 (0.6%)	33 (0.7%)	0.5
Presenting Symptoms	Asymptomatic	4,734 (54%)	2,595 (54%)	2,139 (54%)	**<0.001**
	Atypical Angina	2,123 (24%)	993 (21%)	1,130 (29%)	
	Non-Anginal	1,328 (15%)	839 (18%)	489 (12%)	
	Typical	553 (6.3%)	347 (7.3%)	206 (5.2%)	
Resting ECG Abnormal		5,296 (62%)	3,526 (74%)	1,770 (47%)	**<0.001**
Stress Type	Exercise	3,761 (41%)	1,987 (42%)	1,774 (40%)	**<0.001**
	Pharmacological	5,431 (58.9%)	2,787 (58.4%)	2,644 (28.7%)	
Clinical Response to Stress	Non-ischemic	6,625 (79%)	3,196 (76%)	3,429 (82%)	**<0.001**
	Equivocal	339 (4.0%)	187 (4.4%)	152 (3.7%)	
	Ischemic or Abnormal	1,420 (15.4%)	842 (17.6%)	578 (13.0%)	
ECG Response to Stress	Negative	5,490 (60%)	2,552 (54%)	2,938 (67%)	**<0.001**
	Borderline or Equivocal	453 (4.9%)	335 (7.0%)	118 (2.7%)	
	Positive	1,061 (12%)	513 (11%)	548 (12%)	
Heart Rate (BPM)	Rest	68 (60, 77)	68 (60, 77)	67 (60, 76)	0.056
	Stress Peak	107 (84, 137)	110 (88, 137)	102 (81, 136)	**<0.001**

Systolic Blood Pressure (mmHg)	Rest	130 (120, 146)	130 (120, 140)	134 (120, 150)	**<0.001**
	Stress Peak	146 (126, 170)	150 (130, 170)	144 (122, 170)	**<0.001**
Diastolic Blood Pressure (mmHg)	Rest	80 (71, 80)	80 (74, 80)	80 (70, 85)	**0.048**
	Stress Peak	80 (70, 80)	80 (70, 80)	78 (70, 84)	0.055
Location	Inpatient	938 (11%)	444 (9.3%)	494 (13%)	**<0.001**
	Outpatient	7,361 (87%)	4,285 (90%)	3,076 (83%)	
	Emergency	193 (2.3%)	43 (0.9%)	150 (4.0%)	

Number (%), median (Interquartile range), bold indicates p<0.05.
BMI, body mass index; BPM, beats per minute; CABG, coronary artery bypass graft; CAD, coronary artery disease; ECG, electrocardiogram; LVH, left ventricle hypertrophy; PCI, percutaneous coronary intervention; PVD, peripheral vascular disease; MI, myocardial infarction; TAVR, transcutaneous aortic valve replacement.

Supplementary Table 3: Hyperparameter search parameters.

Parameter Name	Description	Range
**Dimension Reduction Parameters**		
n_components	dimensionality of ultimate embedding space	3
reducer	model for dimension reduction	UMAP
n_neighbors	controls size of local structure	5, 50, 100*
min_dist	minimum distance between embedded points	0.1, 0.15*, 0.25, 0.99
norm_embedding	Whether to normalize axes of embedding	True*, False
metric	evaluation criteria for distance between points	Euclidean, Minkowski, Canberra, Braycurtis*
**Clustering Parameters**		
cluster_option	method for clustering embedded points	hierarchical,<br>kmeans*,<br>gaussian mixture model
k	sweep range: number of clusters for any cluster_option	2,3*-10

* identifies the optimal parameter used for unsupervised cluster analysis.

Supplementary Table 4: Demographic and imaging characteristics for all patients and clusters in the external cohort.

		All patients	Cluster 1	Cluster 2	Cluster 3	P
N		4,447	1,799	2,213	435	-
Age (years)		68 (60, 76)	65 (57, 71)	71 (63, 77)	72 (64, 79)	**<0.001**
Male		3,345 (75%)	1,480 (82%)	1,586 (72%)	279 (64%)	**<0.001**
BMI (kg/m^2)		28 (25, 31)	28 (25, 31)	27 (25, 31)	29 (26, 33)	**<0.001**
Hypertension		3,746 (84%)	1,433 (80%)	1,930 (87%)	383 (88%)	**<0.001**
Diabetes Mellitus		1,530 (34%)	521 (29%)	811 (37%)	198 (46%)	**<0.001**
Dyslipidemia		2,885 (65%)	1,083 (60%)	1,536 (70%)	266 (61%)	**<0.001**
Family History of CAD		1,929 (43%)	901 (50%)	934 (42%)	94 (22%)	**<0.001**
Smoking		1,950 (44%)	721 (40%)	1,096 (50%)	132 (30%)	**<0.001**
Previous MI		2,480 (56%)	961 (53%)	1,312 (59%)	207 (48%)	**<0.001**
Previous Cardiac Surgery or Intervention	PCI	2,697 (61%)	1,145 (64%)	1,299 (59%)	253 (58%)	**0.004**
	CABG	252 (6.7%)	115 (8.1%)	112 (5.8%)	25 (5.9%)	**0.029**
	TAVR	3 (<0.1%)	0 (0%)	3 (0.1%)	0 (0%)	0.2
	Other	33 (0.7%)	4 (0.2%)	25 (1.1%)	4 (0.9%)	**0.004**
Presenting Symptoms	Asymptomatic	2,139 (54%)	855 (53%)	1,179 (59%)	105 (29%)	**<0.001**
	Atypical Angina	1,130 (29%)	458 (28%)	453 (23%)	219 (62%)	
	Non-Anginal	489 (12%)	214 (13%)	244 (12%)	31 (8.7%)	
	Typical	206 (5.2%)	84 (5.2%)	121 (6.1%)	1 (0.3%)	
Resting ECG Abnormal		1,770 (47%)	774 (47%)	913 (46%)	83 (59%)	**0.017**
Stress Type	Exercise	1,774 (40%)	1,749 (97%)	16 (0.7%)	9 (2.1%)	**<0.001**
	Pharmacological	2,644 (59%)	49 (2.7%)	2,169 (99%)	426 (98%)	
Clinical Response to Stress	Non-ischemic	3,429 (82%)	1,359 (78%)	1,884 (87%)	186 (72%)	**<0.001**
	Equivocal	152 (3.7%)	70 (4.0%)	47 (2.2%)	35 (13%)	
	Ischemic or Abnormal	578 (13%)	309 (18%)	230 (10.7%)	39 (15%)	
ECG Response to Stress	Negative	2,938 (67%)	974 (54%)	1,789 (82%)	175 (40%)	**<0.001**
	Borderline or Equivocal	118 (2.7%)	80 (4.4%)	16 (0.7%)	22 (5.1%)	
	Positive	548 (12%)	449 (25%)	75 (3.4%)	24 (5.5%)	
Heart Rate (BPM)	Rest	67 (60, 76)	69 (60, 79)	65 (58, 75)	69 (61, 77)	**<0.001**
	Stress Peak	102 (81, 136)	139 (129, 150)	83 (73, 96)	88 (77, 101)	

Systolic Blood Pressure (mmHg)	Rest	134 (120, 150)	130 (120, 144)	135 (122, 150)	140 (125, 154)	**<0.001**
	Stress Peak	144 (122, 170)	170 (150, 184)	128 (114, 144)	137 (117, 159)	
Diastolic Blood Pressure (mmHg)	Rest	80 (70, 85)	80 (70, 84)	80 (70, 85)	76 (69, 83)	**<0.001**
	Stress Peak	78 (70, 84)	82 (78, 90)	72 (65, 80)	72 (62, 80)	
Location	Inpatient	494 (13%)	117 (7.9%)	330 (17%)	47 (15%)	**<0.001**
	Outpatient	3,076 (83%)	1,323 (90%)	1,536 (79%)	217 (71%)	
	Emergency	150 (4.0%)	33 (2.2%)	74 (3.8)	43 (14%)	
Dose (MBq)	Rest	142 (7, 160)	149 (0, 155)	150 (18, 166)	18 (8, 19)	**<0.001**
	Stress	185 (25, 450)	185 (28, 450)	185 (29, 492)	9 (8, 23)	**<0.001**

Number (%), median (Interquartile range), bold indicates p<0.05.
BMI, body mass index; BPM, beats per minute; CABG, coronary artery bypass graft; CAD, coronary artery disease; ECG, electrocardiogram; LVH, left ventricle hypertrophy; PCI, percutaneous coronary intervention; PVD, peripheral vascular disease; MI, myocardial infarction; TAVR, transcutaneous aortic valve replacement.

Supplementary Table 5: Quantitative imaging features for all clusters in the internal cohort.

	**Cluster 1**	**Cluster 2**	**Cluster 3**	**P value Cluster 1 vs 3**
N	2,005	1,580	1,189	-
Percent Ischemia (%)	2.8 [1.0, 5.7]	4.4 [2.3, 7.2]	2.8 [0.6, 6.8]	0.69
Stress Total Perfusion Deficit (%)	3.9 [1.4, 9.9]	6.4 [2.8, 14.2]	5.2 [2.0, 13.0]	**<0.001**
Rest Total Perfusion Deficit (%)	0.5 [0.0, 3.7]	1.2 [0.0, 6.9]	2.9 [0.7, 8.3]	**<0.001**
Stress End Diastolic Volume (ml)	85.9 [69.5, 107.0]	96.5 [75.9, 122.9]	87.5 [66.0, 119.3]	0.103
Stress End Diastolic Wall Volume (ml)	136.5 [121.1, 154.9]	138.8 [122.2, 161.0]	144.6 [123.4, 173.2]	**<0.001**
Stress End Diastolic Length (mm)	75.5 [69.9, 81.2]	77.1 [70.7, 83.2]	72.2 [64.6, 80.1]	**<0.001**
Stress Shape Index End Diastolic (%*0.01)	0.61 [0.57, 0.66]	0.64 [0.60, 0.69]	0.67 [0.61, 0.73]	**<0.001**
Stress End Systolic Volume (ml)	35.2 [25.1, 50.8]	41.9 [28.8, 61.9]	36.9 [23.8, 62.1]	**0.020**
Stress End Systolic Wall Volume (ml)	136.4 [121.1, 155.0]	138.8 [122.2, 161.0]	144.8 [123.3, 173.3]	**<0.001**
Stress End Systolic Length (mm)	68.6 [62.5, 74.9]	70.5 [63.6, 78.0]	66.0 [57.8, 74.4]	**<0.001**
Stress Shape Index End Systolic (%*0.01)	0.46 [0.42, 0.51]	0.48 [0.43, 0.54]	0.51 [0.45, 0.59]	**<0.001**
Stress Ejection Fraction (%)	58.6 [51.7, 64.4]	55.9 [47.2, 63.4]	57.1 [46.6, 65.4]	**0.0016**
Stress Thickening Abnormal Extent (%)	0.16 [0.0, 7.0]	2.6 [0.0, 14.4]	1.8 [0.0, 16.7]	**<0.001**
Stress Motion Abnormal Extent (%)	1.4 [0.0, 15.3]	4.1 [0.1, 27.8]	7.5 [0.4, 31.4]	**<0.001**
Stress Average Motion (mm)	6.7 [5.9, 7.5]	6.7 [5.6, 7.6]	6.4 [5.3, 7.5]	**<0.001**
Rest Ejection Fraction (%)	59.7 [52.3, 65.9]	59.5 [50.9, 67.1]	57.6 [45.7, 67.0]	**<0.001**
Rest End Diastolic Volume (ml)	90.8 [74.0, 111.6]	96.1 [76.3, 122.7]	78.0 [59.6, 108.6]	**<0.001**
Rest End Systolic Volume (ml)	36.1 [25.8, 51.6]	38.6 [25.9, 57.3]	33.0 [20.7, 56.5]	**0.020**
Transient Ischemic Dilation	0.95 [0.88, 1.0]	1.0 [0.94, 1.1]	1.0 [0.95, 1.1]	**<0.001**

Number (%), median (Interquartile range), bold indicates p<0.05.

Supplementary Table 6: Cardiovascular outcomes for all patients and clusters in the external cohort.

	**All patients**	**Cluster 1**	**Cluster 2**	**Cluster 3**	**P**
N	4,447	1,799	2,213	435	
Early revascularization *	401 (11%)	172 (13%)	176 (9.3%)	53 (15%)	**<0.001**
Revascularization	551 (15%)	233 (16%)	222 (11%)	95 (25%)	**<0.001**
Myocardial infarction	201 (5.6%)	52 (3.9%)	105 (5.6%)	44 (12%)	**<0.001**
Unstable angina	118 (4.4%)	48 (4.3%)	64 (4.2%)	6 (40%)	**<0.001**
PCI	243 (6.4%)	76 (5.3%)	112 (5.8%)	55 (13%)	**<0.001**
CABG	252 (6.7%)	115 (8.1%)	112 (5.8%)	25 (5.9%)	**0.029**
MACE	1,063 (24%)	365 (20%)	549 (25%)	149 (34%)	**<0.001**
All-cause mortality	312 (7.0%)	52 (2.9%)	206 (9.3%)	54 (12%)	**<0.001**

Number (%), bold indicates p<0.05.
* 90 days.
CABG, coronary artery bypass graft; MACE, major adverse cardiovascular events. PCI, percutaneous coronary intervention.

Supplementary Table 7: Disaggregation of MACE survival by gender in the internal cohort.

Cohort subset	Cluster 1		Cluster 2		Cluster 3		Global Log-Rank Significance
	N	Hazard Ratio	N	Hazard Ratio	N	Hazard Ratio	
**All** (n=4,774)	2,005	reference	1,580	1.5 [1.4-1.7]	1,189	1.7 [1.5-1.9]	2.3e-17
**Males** (n=3,704)	1,667	reference	1,264	1.5 [1.3-1.8]	773	1.7 [1.5-1.9]	1.4e-14
**Females** (n=1,070)	338	reference	316	1.6 [1.2-2.2]	416	1.9 [1.5-2.6]	1.3e-5

Supplementary Table 8: Disaggregation of all-cause mortality survival by gender in the internal cohort.

Cohort subset	Cluster 1		Cluster 2		Cluster 3		Global Log-Rank Significance
	N	Hazard Ratio	N	Hazard Ratio	N	Hazard Ratio	
**All** (n=4,774)	2,005	reference	1,580	3.1 [2.4-3.9]	1,189	4.7 [3.7-5.9]	4.5e-47
**Males** (n=3,704)	1,667	reference	1,264	2.8 [2.2-3.7]	773	4.5 [3.5-5.8]	2.2e-34
**Females** (n=1,070)	338	reference	316	4.8 [2.5-9.4]	416	7.0 [3.7-13.0]	8.9e-14

Supplementary Table 9: Disaggregation of MACE survival by gender in the external cohort.

Cohort subset	Cluster 1		Cluster 2		Cluster 3		Global Log-Rank Significance
	N	Hazard Ratio	N	Hazard Ratio	N	Hazard Ratio	
**All** (n=4,447)	1,799	reference	2,213	1.2 [1.1-1.4]	435	4.2 [3.4-5.1]	9.9e-37
**Males** (n=3,345)	1,480	reference	1,586	1.2 [1.0-1.4]	279	4.2 [3.3-5.3]	6.8e-25
**Females** (n=1,102)	319	reference	627	1.6 [1.1-2.2]	156	5.4 [3.6-7.9]	3.4e-15

Supplementary Table 10: Disaggregation of all-cause mortality survival by gender in the external cohort.

Cohort subset	Cluster 1		Cluster 2		Cluster 3		Global Log-Rank Significance
	N	Hazard Ratio	N	Hazard Ratio	N	Hazard Ratio	
**All** (n=4,447)	1,799	reference	2,213	3.3 [2.5-4.5]	435	5.9 [4.0-8.6]	8.9e-24
**Males** (n=3,345)	1,480	reference	1,586	3.3 [2.3-4.7]	279	6.1 [3.9-9.6]	1.0e-17
**Females** (n=1,102)	319	reference	627	3.2 [1.7-6.1]	156	5.2 [2.5-10.8]	6.0e-06

Supplementary Table 11: MACE survival by site in the internal cohort.

Internal Cohort Site	Assuta		Brigham and Women's		Cedars-Sinai		Oregon		Global Log-Rank Significance
	N	Hazard Ratio	N	Hazard Ratio	N	Hazard Ratio	N	Hazard Ratio	
**Patients** (n=4,774)	2,773	reference	759	0.7 [0.6-0.9]	803	1.2 [1.0-1.3]	439	1.2 [1.0-1.4]	2.5e-7

Supplementary Table 12: All-cause mortality survival by site in the internal cohort.

Internal Cohort Site	Assuta		Brigham and Women's		Cedars-Sinai		Oregon		Global Log-Rank Significance
	N	Hazard Ratio	N	Hazard Ratio	N	Hazard Ratio	N	Hazard Ratio	
**Patients** (n=4,774)	2,773	reference	759	0.7 [0.4-1.3]	803	1.7 [1.1-2.7]	439	2.0 [1.3-3.1]	1.5e-4
